# KID - an algorithm for fast and efficient text mining used to automatically generate a database containing kinetic information of enzymes

**DOI:** 10.1186/1471-2105-11-375

**Published:** 2010-07-13

**Authors:** Stephanie Heinen, Bernhard Thielen, Dietmar Schomburg

**Affiliations:** 1 Max Planck Institute for Neurologic Research, Gleuelerstr. 50, 50931 Cologne, Germany; 2 Department of Biochemistry, University of Cologne, Zuelpicherstr. 47, 50674 Cologne, Germany; 3 Stieglitzweg 20, 50829 Cologne, Germany; 4 Dpt. Of Bioinformatics & Biochemistry, University of Braunschweig, Institute of Technology, Langer Kamp 19B, 38106 Braunschweig, Germany

## Abstract

**Background:**

The amount of available biological information is rapidly increasing and the focus of biological research has moved from single components to networks and even larger projects aiming at the analysis, modelling and simulation of biological networks as well as large scale comparison of cellular properties. It is therefore essential that biological knowledge is easily accessible. However, most information is contained in the written literature in an unstructured way, so that methods for the systematic extraction of knowledge directly from the primary literature have to be deployed.

**Description:**

Here we present a text mining algorithm for the extraction of kinetic information such as K_M_, K_i_, k_cat _etc. as well as associated information such as enzyme names, EC numbers, ligands, organisms, localisations, pH and temperatures. Using this rule- and dictionary-based approach, it was possible to extract 514,394 kinetic parameters of 13 categories (K_M_, K_i_, k_cat_, k_cat_/K_M_, V_max_, IC_50_, S_0.5_, K_d_, K_a_, t_1/2_, pI, n_H_, specific activity, V_max_/K_M_) from about 17 million PubMed abstracts and combine them with other data in the abstract.

A manual verification of approx. 1,000 randomly chosen results yielded a recall between 51% and 84% and a precision ranging from 55% to 96%, depending of the category searched.

The results were stored in a database and are available as "KID the KInetic Database" via the internet.

**Conclusions:**

The presented algorithm delivers a considerable amount of information and therefore may aid to accelerate the research and the automated analysis required for today's systems biology approaches. The database obtained by analysing PubMed abstracts may be a valuable help in the field of chemical and biological kinetics. It is completely based upon text mining and therefore complements manually curated databases.

The database is available at http://kid.tu-bs.de. The source code of the algorithm is provided under the GNU General Public Licence and available on request from the author.

## Background

The availability of a number of different OMICS technologies has made it possible that - in addition to the traditional molecular biology methods - whole "systems", from molecular networks via cells and organs to whole organisms have become the focus of large scale research projects in all biosciences. Whereas it is still possible to manually follow the literature in a certain limited area the rapid growth of scientific literature does not allow to e.g. extract the information on all enzymes in a certain organism from the literature in a sensible time, or to make large scale comparisons between the metabolic functions of different organisms. Moreover in areas of drug development the knowledge on binding properties between enzyme and ligand is essential [1].

Several databases are available providing information about enzymes and their characteristics like e.g. BRENDA [2-4] with currently 92,291 entries for K_M_, 32,484 for k_cat_, 21,833 for K_i _and 33,372 for specific activity [2], Kinetikon [5], KMedDB [6], KDBI [7], DOQCS [8], SABIO-RK [9] and IUPAC-kinetic [10], respectively. However, these databases are far from complete, forcing scientists to a time consuming manual extraction of values from the literature if a systematic research approach is followed.

One approach for a faster and simpler access to this information is to use text mining [11-14], i.e. the computer aided extraction of data from natural written text [1,15-17]. Current algorithms include machine learning (e.g. Kinetikon [5]), statistic (e.g. FRENDA and AMENDA [3]), rule-based (KiPar [18] and BioRAT [19]) and mixed approaches (SUISEKI [20]).

Here we present a rule- and dictionary-based [1,15] text mining algorithm for the extraction of kinetic data, developed with a focus on a fast calculation time and a high precision of the received information.

The attained kinetic enzyme information is stored and presented in the database "KID the Kinetic Database", which contains information extracted from about 17 million PubMed [21] abstracts.

## Construction and content

### Data basis

The dictionaries used were mostly generated automatically using public databases and complemented manually (see table 1). Names for organisms were extracted from NCBI Taxonomy [22] and BRENDA [2]. The same applies to the dictionaries for enzyme names (including unique synonyms), EC numbers and ligands. Expressions for tissues were collected partially from the "BRENDA tissue ontology" [3] and UniProt and supplemented manually. Since an identification of an enzyme can be obtained by its name or the EC number, these categories are combined and referred to as enzyme in the following. The numbers of items each category comprises is given in table 1.

**Table 1:** Dictionaries used for identifying entities in the text and their size.

Category	Number of entries
binding phrases	2,068

ligands	666,563
organisms	506,698
enzyme-names	52,179
EC numbers	9,035
tissues	8,512

expressions for K_M_	321
expressions for K_i_	77
expressions for K_d_	76
expressions for k_cat_	76
expressions for IC_50_	75
expressions for V_max_	74
expressions for n_H_	69
expressions for t_1/2_	64
expressions for k_cat_/K_M_	42
expressions for pI	31
expressions for K_a_	28
expressions for S_0.5_	22
expressions for specific activity	14
expressions for V_max_/K_M_	12
expressions für pH	12
expressions for temperature	6

units for V_max_	325
units for specific activity	176
concentrations	116
units for k_cat_/K_M_	71
units for t_1/2_	31
units for k_cat_	31
units for V_max_/K_M_	27
units for K_a_	18

Since one term can only be mapped to one category (see below), ambiguous terms have to be assigned to one dictionary or excluded from the search. For example "IPP" is an acronym used for an enzyme (inositol-1,4-bisphosphate 1-phosphatase) as well as for a ligand (isopentenyl diphosphate).

Furthermore, dictionaries for different identifiers and numerical expressions of K_M_, K_i_, k_cat_, k_cat_/K_M_, V_max_, IC_50_, S_0.5_, K_d_, K_a_, t_1/2_, pI, n_H_, V_max_/K_M _and specific activity (including a number of typing errors frequently found) together with their units are collected manually (see table 2). Each of them is tokenized according to the mechanism mentioned below.

**Table 2:** Extract from the dictionary for K_M_.

**Synonym for K**_**M**_	
Michaelis constant	k(m)
Michaelis constants	k(m, app)
Michaelis-Menten constant	k(m)app
Michaelis-Menten constants	k(m), app
Michaelis constant(km)	km ap
Michaelis-constant (km)	k-m
affinity constant (km)	Kappm

16,953,021 PubMed [21] abstracts available in 2007 were analysed. Each abstract is split into sentences when a dot followed by a whitespace is detected. Notable exceptions to this rule are recognized abbreviations like "i.v." or "e.g.". The sentences are hereafter translated into token [16] by splitting at whitespace.

### Algorithm

The algorithm is divided into two parts, the identification of entities in the text (i.e. tagging [13]) and a rule-based linkage of these units.

**Figure 1:**
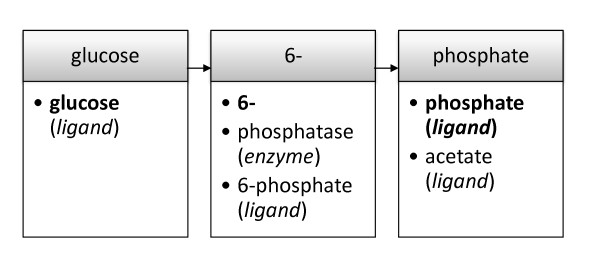
**Order of the identification of a textual phrase with the hash-based dictionary-structure**. The sequence of token in the sentence is shown in grey boxes. Corresponding token found in the dictionary are listed below in white boxes. The identified sequence is marked in bold letters and identical with the sequence of token found in the sentence. Categories are denoted in brackets below the entity.

In order to provide a fast identification of entities in the text a data structure utilizing hash tables was generated (see figure 1). Starting from the first word (in case of figure 1 "glucose"), different series of subsequent tokens form a named entity, e.g. "glucose phosphatase" or "glucose 6- phosphate". The last token of a sequence is marked by a flag describing the corresponding category, e.g. *enzyme *and *ligand *for "glucose phosphatase" and "glucose 6- phosphate", respectively.

For the process of identification the tokens in a sentence are examined for their existence within the hash one by one, starting from a token like "glucose" and proceeding with the following words. The longest sequence of tokens with a flag on the last token is accepted and the combination is then marked as a phrase carrying the associated flag.

Numbers are recognized in a second step using regular expressions in sentences where previously a kinetic expression has been found. If a number is followed by a kinetic unit, then both are combined to a common phrase. If certain phrases, for example a unit which does not match the kinetic expression, are found right behind the number, it is removed, which reduces the risk of incorrect linkage.

If ligands are following a negation phrase like e.g. "in absence of ATP", the ligand is also removed.

**Figure 2:**
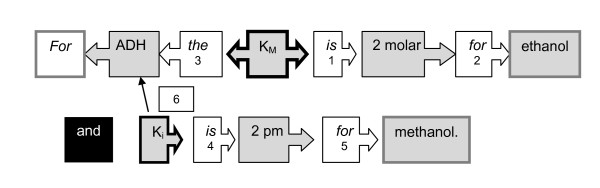
**Scheme of the linkage of an exemplary sentence**. Gray boxes symbolise an entity of a category. White boxes represent binding phrases written in cursive letters. The kinetic expressions (K_M_, K_i_) are used as initial anchor for the linkage. a) The first binding word links K_M _to a molar concentration (1). Next to this concentration another binding word is found which links the ligand (2). Further to the right, no entity of another category or binding phrase can be found and the search is stopped. It is searched to the left side of the kinetic expression, linking it with an enzyme through another binding phrase (3). At the beginning of the sentence the search is stopped. b) K_i _is linked to a concentration with the help of a binding phrase (4). Then the concentration is connected with a ligand (5) and the search is stopped at the end of the sentence. Due to the missing linkage with an enzyme the kinetic expression is linked to the only enzyme name in this sentence (6).

For the linkage of identified entities a direct or an indirect mechanism is used alternatively. The **direct linkage **takes place if two entities are found in direct neighbourhood or if two entities of different categories are combined by certain predefined phrases (see figure 2). The starting point of the linkage is the position of the kinetic expression (e.g. K_M_). From this position a direct linkage will firstly take place to the right side following the natural reading direction for english written text. If a linkage to another entity occurs, the position of this entity will be taken as a new starting point for the next linkage. The linkage is stopped if no binding phrase is identified or no further entity of a not yet filled group is found. Additional reasons for a stop are the end of the sentence or if a comma, which is not followed by a keyword like "which" is found. The position of the kinetic expression is then used for a search to the left side, which follows the same mechanism as explained.

A special treatment is applied for enumerations, i.e. if more than one entity of the same category is found in one sentence, being separated by commas and "or" or "and" between the last two entities. These terms are mapped one after the other to the entities of the remaining categories.

In sentences which contain more than one enumeration the collections with the largest number of terms are linked sequentially. I.e. in the phrase "..results for enzyme a and enzyme b with ligand c and ligand d.." enzyme a will be linked to ligand c and enzyme b will be linked to ligand d. This linkage of enumerations is not applied to kinetic categories.

If one of the categories cannot be filled by direct linkage, an **indirect linkage **takes place. It is checked whether one and only one entry of the corresponding category is present in the sentence and if so, this entry is accepted. If the indirect search over the sentence is not successful, an indirect search over the abstract, followed by a search over the title, will be carried out with the same mechanism. The indirect linkage is not performed for pH and temperature on level of the title and the abstract, since the entities of these categories are numbers not marked by a unit and can therefore not be distinguished from numbers not related to the kinetic constant. During indirect linkage on level of the sentence the missing unit helps to isolate the number from the one belonging to the kinetic category.

In the case that an enzyme name is found but no according EC number (or the opposite), it is checked if this information can be added automatically with a query in BRENDA [2].

## Utility

### Distribution of linkages

**Figure 3:**
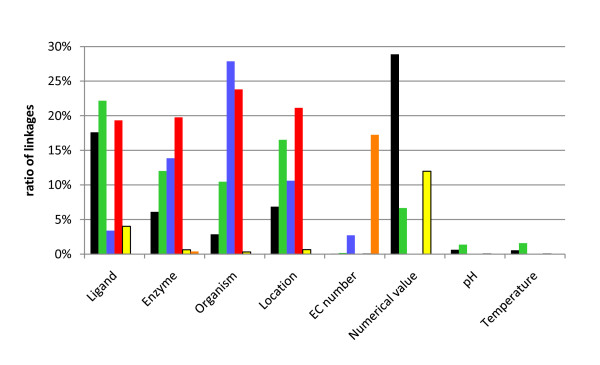
**Distribution of linkage types for the extracted kinetic categories**. The ratio of linked terms of the kinetic category shown on the x-axis is referred to the total number of kinetic expressions (514,394) (black bars: direct linkage; green bars: indirect linkage on the level of the sentence; blue bars: indirect linkage on the level of the abstract; red bars: indirect linkage on the level of the title; yellow bars: listing; orange bars: automatically completed from BRENDA [2]).

Figure 3 shows the distribution of linkage types summarized for the kinetic categories. A list of distributions subdivided into the kinetic categories is available as additional file 1. The majority of ligands (22% of the total number of extracted kinetic parameters) are indirectly linked on the level of the sentence, whereas 18% of the ligands are linked by direct linkage. The indirect linkage on level of the title and the extraction from listings is carried out with 19% and 4%, respectively.

For enzyme names most of the results are linked indirectly on the title level (20%). The indirect linkage on level of the sentence and on level of the abstract is carried out in 12% and 14% of the cases, respectively. A smaller amount (6%) is linked directly.

Concerning the organisms, the indirect linkage on the abstract level (27%) and on the level of the title (24%) is used most often. Approximately 10% are linked indirectly on the level of the sentence.

For the localisation, the linkage mainly takes place on level of the title (21%) and the abstract (11%). 17% are linked by the use of the indirect linkage on the sentence level.

The majority of the EC numbers are annotated automatically from BRENDA (17%), a small number is linked on the level of the abstract (3%).

For numerical values the direct assignment is mainly applied with a ratio of 29%. 12% of the values are extracted from listings and in 7% of the cases the linkage takes place indirectly on level of the sentence.

Comparatively few values are linked for pH and temperature. 1.3% and 1.6% are linked indirectly on the sentence level, respectively.

Summarizing, the direct linkage is successful for linking numbers (29% of linked numbers) and ligands (18% of linked ligands), whereas the indirect linkage is used for the linkage of ligands (22% on the level of the same sentence), organism and localisation (24% and 21% on the level of the title and 28% and 11% on the level of the abstract, respectively) and enzyme names (22% on the level of the title).

Using the linkage it was possible to generate more than one result per abstract for 46.5% of the abstracts containing kinetic information.

### Content of the database

**Figure 4:**
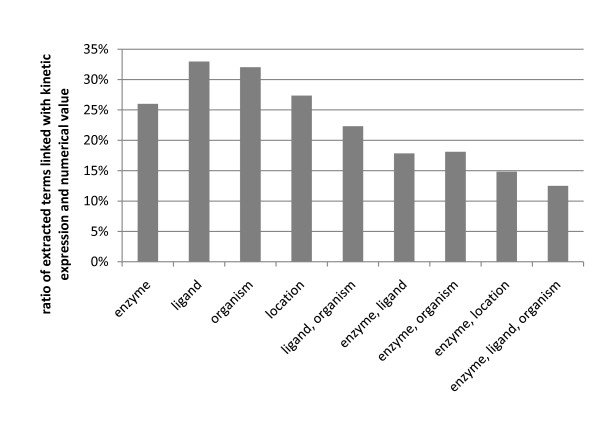
**Quantitative distribution of categories linked with a kinetic expression and its numerical value**. On the y-axis the ratio (in comparison to the total number of results) of extracted values of the corresponding category or a combination of categories is shown.

469,113 kinetic parameters are found in 260,316 abstracts, with an over-representation of IC_50_, t_1/2 _and K_M _(see table 3). After itemizing the enumerations, the number increases to 514,394 parameters. 244,523 kinetic expressions are linked to a numeric value (48%). About 53% (271,256) of the entries were associated to an enzyme, 66% (342,003) to a ligand and 65% (335,854) to an organism, respectively. When including numerical values, the numbers decrease to 26%, 33% and 32%, respectively (see figure 4). A value can be connected to an enzyme and a ligand in 18% of the cases, an enzyme and an organism also in 18%. In 13%, the three categories enzyme, organism and ligand were linked to a numerical value. A detailed list of linkages is available as additional file 2 and additional file 3. For a comparison of the extent of covered abstracts with KMedDB [6] and BRENDA [3] see table 4.

**Table 3:** Amount of extracted entities for the corresponding kinetic categories.

Kinetic category	Amount
IC_50_	105,240
t_1/2_	91,429
K_M_	74,253
K_d_	65,401
K_i_	42,344
spezific activity	37,001
V_max_	36,862
K_a_	18,903
pI	17,481
n_H_	5,465
k_cat_/K_M_	5,242
S_0.5_	2,899
V_max_/K_M_	1,469

**Table 4:** Comparison of the content from different databases providing kinetic information.

Kinetic expression	KID (PubMed IDs/entries)	**KMedDB **[6]**(PubMed IDs)**	**BRENDA **[3]**(PubMed IDs/entries)**
t_1/2_	57,658/91,429	49,608	-
IC_50_	54,938/105,240	28,709	1,292/8,473
K_M_	45,896/74,253	39,719	18,571/92,291
K_d_	40,244/65,401	39,448	-
V_max_	23,985/36,862	29,851	-
K_i_	22,805/42,344	20,683	4,214/21,833
k_cat_	7,325/10,405	9,528	5,492/32,484

### Evaluation of the database

**Figure 5:**
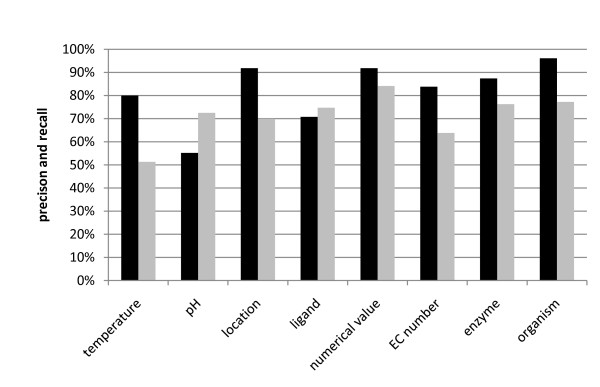
**Precision and recall of the categories used during extraction**. Precision shown in black bars and recall in grey bars.

In order to evaluate the performance of the algorithm, precision and recall [13,15,17] from a number of 1,002 entries in the results (from 510 randomly chosen PubMed IDs, see additional file 4; The abstracts were chosen by creating random numbers using a C++ implementation of the Mersenne Twister pseudo-random number generator [23]) were manually reviewed. A recall of 51% to 84% and a precision of 55% to 96% could be achieved depending on the category (see figure 5). The recall for BioRAT [19], a database built using a rule-based approach, is published to be 22%, whereas the precision is denoted to be 55% [19]. FRENDA and AMANDA reach a precision of 65% and 76% and a recall of 72% and 11%, respectively [3].

Breaking down the precision into types of linkages results in an overall precision of 91%, 78%, 89% and 88% for direct linkage, indirect linkage on the sentence level, abstract level and title level, respectively (compare additional file 5). A notable discrepancy becomes apparent when examining the precision of ligands, which is 86% for direct linkage, but averagely 62% for indirect linkage.

**Figure 6:**
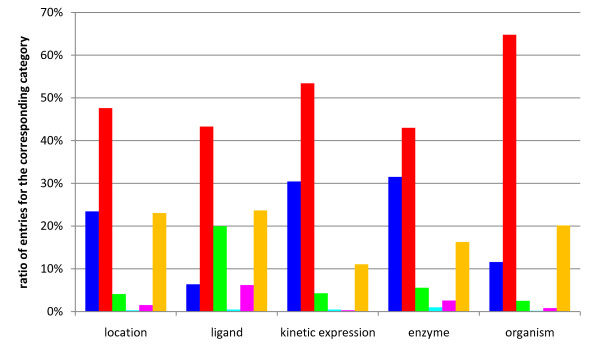
**Classification of information contained in abstracts with reference to the performance of the algorithm**. Categories are shown on the x-axis, while the ratio of abstracts for the corresponding category are shown on the y-axis (blue bars: none in abstract; red bars: correctly linked; green bars: wrong and a right one available; cyan bars: wrong and no right in abstract; magenta bars: incomplete but not incorrect; orange bars: missing in results).

Furthermore, the 1,002 entries were categorized as followed: none in abstract; correctly linked; wrong and a right one available; wrong and no right in abstract; incomplete but not incorrect; missing in results (compare figure 6; temperature, pH and EC number are not included into the figure since in more than 90% of the cases no entry is contained in the abstract). Here, "incomplete but not incorrect" marks entries like e.g. when reductoisomerase is tagged instead of 1-deoxy-D-xylulose 5-phosphate reductoisomerase (PubMed ID 10787409). Several reasons for wrong or missing entities were identified: In case of wrong entities, abbreviations like e.g. KA used for "kainic acid" (PubMed ID 10718304) are a major cause for false positives. Similar applies for synonyms like e.g. KI as molecular formula of potassium iodide instead of inhibitory constant (e.g. PubMed ID 1289838). Missing entries in the dictionaries are a reason for them also missing in the results (e.g. "wheat Triticum aestivum" not found in the title of PubMed ID 14607490). In other cases the algorithm was not capable to assign a correct entity via the indirect linkage since more than one entry was found (like in PubMed ID 9430617, where "bacterial" and "Xenopus" are identified on the level of the abstract but are not linked).

### Calculation time

The algorithm was implemented in C++ together with the Qt4 framework [24]. Kinetic information from 16,953,021 PubMed abstracts (2007) [21] was extracted with a single core application within about 18 hours using a computer with an AMD Turion X2-TL-52 processor with 1.6 GHz. During this time, a maximum of approx. 300 MB RAM was occupied (mainly by the dictionaries).

**Figure 7:**
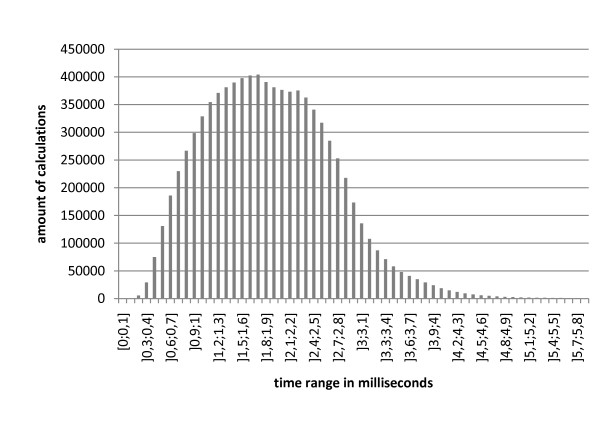
**Distribution of calculation times for identification of abstracts without kinetic content**. The amount of calculations covered within the time interval (given on the x-axis) is shown on the y-axis.

**Figure 8:**
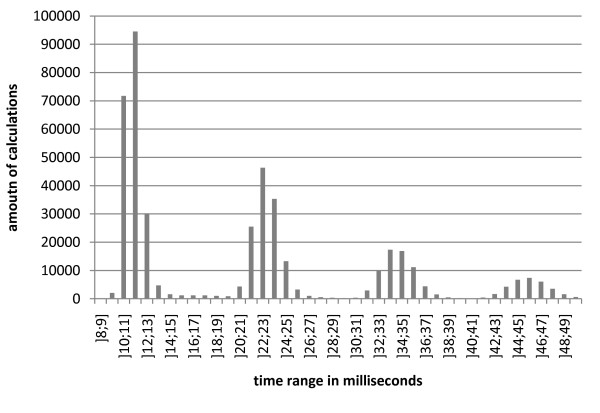
**Distribution of calculation times for identification of abstracts with kinetic content**. The amount of calculations covered within a given time interval is shown on the y-axis.

When no kinetic category is found, the identification of numbers via regular expressions and the linkage is skipped, leading to an identification time of averagely 1.9 ms ± 0.8 ms (standard deviation; compare figure 7). For abstracts containing kinetic information, several maxima can be distinguished with an average calculation time of 24 ms (figure 8). The average time for the subsequent linkage is 0.04 ms.

### Range and coverage of the extracted data

**Figure 9:**
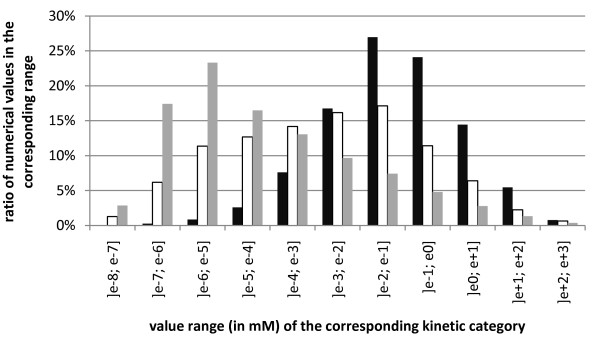
**Range of extracted values for K_M_, K_i_and K_d_**. On the x-axis the ranges of the numerical values of K_M _(black), K_i _(white), K_d _(grey) are shown, whereas the y-axis shows the number of kinetic values in the corresponding interval compared to the total number of extracted values.

Distribution maxima of the values for K_M_, K_i _and K_d _are found between 10^-2 ^to 10^0 ^millimolar for K_M_, 10^-3 ^to 10^-1 ^millimolar for K_i _and 10^-7 ^to 10^-4 ^millimolar for K_d_, respectively (see figure 9).

**Figure 10:**
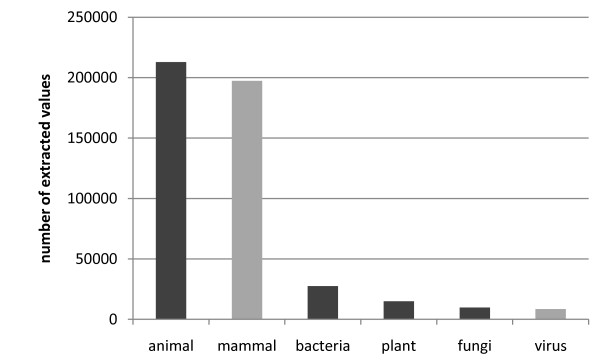
**Classification of extracted organisms linked with kinetic information**. Dark grey bars denote kingdoms, light grey bars are organismns of other (sub)groups.

63% of the 335,854 organisms that could be linked to kinetic information belong to animals (212,929; see figure 10). The second largest group are bacteria (27,497), while the remaining groups were represented in smaller numbers. The eight most often extracted organisms are listed in table 5.

**Table 5:** Most often extracted organisms in all kinetic categories.

Organism	Amount
human	86,338
rat	56,456
mouse	15,368
cattle	11,379
rabbit	11,360
pork	9,326
escherichia coli	9,205
yeast	4,641

### Online access to the database

**Figure 11:**
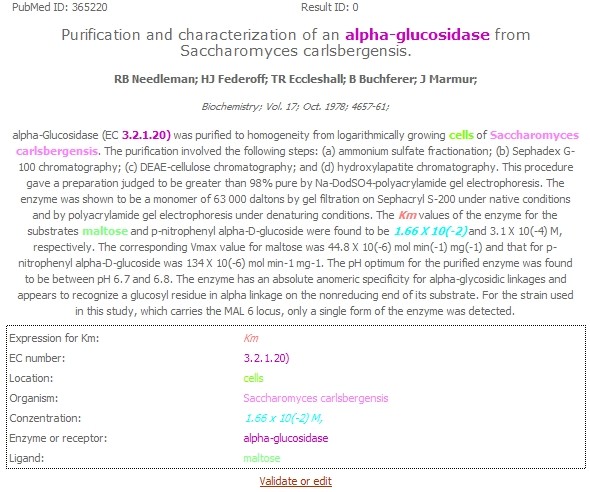
**Presentation of the results inside the web presence**. The original abstract of PubMed ID 365220 is shown. Screenshot taken from the web interface available under http://kid.tu-bs.de.

For the public access of the database a query system based on the Joomla CMS [25] named "KID the KInetic Database" is available. The user can perform a filtered query to search the desired information from the database. The results are then presented in a table, from which the user can move to the original abstract, where the results are marked by colour (see figure 11).

## Discussion

### Comparison of the algorithm

Since the algorithm is dictionary-based, the quality of the identification is limited by the amount and quality of the entries in the dictionaries. By transforming the entries in the dictionaries into small letters, misspellings due to an incorrect use of small and tall letters can be avoided, in contradiction to increasing ambiguity. Considering e.g. the extraction of ligands, where about 20% are falsely linked (see figure 6), more entries in the dictionary will not lead to better results, since a wrong entry has already been linked. Removing false positives from the dictionary will by contrast negatively impact on recall.

However, this kind of algorithm has certain advantages compared to others, e.g. those utilizing machine learning [26]. These are mainly based on Hidden Markov Models [15,17] using a reading horizon of e.g. three tokens, i.e. a linkage of entities can only be recognized if these are contained within the first three tokens, which slows the recognition of long range relations.

The published algorithms for the development of FRENDA and AMANDA are mainly based on co-occurrences [3]. This concept is based on a statistical significance for linked entities, i.e. pairs of entities often found in direct neighbourhood or within a certain range are linked together. Hence, in contrast to the algorithm in this paper, it is e.g. not possible to gather numbers because of their varying nature.

For the linkage of numbers distinct and explicit values like e.g. 2 molar are necessary. Indirectly mentioned values like "... a value that is 50 times higher than the K_M _for this substrate." [27] will therefore not be recognized. However, we are not aware of an algorithm that is capable of gathering such numerical values from sentences.

### Characteristics of the database

The comparison of the PubMed IDs manually extracted in BRENDA shows an overlap with KID of 565 for IC_50_, 9,055 for K_M_, 2,219 for K_i _and 2,694 IDs for k_cat_, respectively; i.e. about half of the IDs covered in BRENDA are also contained in KID. The ratio of kinetic constant entries to PubMed IDs is higher in BRENDA (about 4 to 1 compared to 2 to 1), which is to a certain extent caused by the fact that whole articles instead of abstract are evaluated.

A further comparison of 100 randomly chosen abstracts which are contained in KID but not in BRENDA reveals, that in 70 cases information was extracted correctly and is not available in BRENDA (see additional file 6). 27 abstracts contain information which was correctly recognized, but is not within the scope of BRENDA, since e.g. a tissue instead of an explicit enzyme is mentioned. The remaining 3 abstracts were false positives, e.g. caused by the use of KM as an abbreviation for "Krushinsky-Molodkina" strain of rats in PubMed ID 6,538,738.

Examining 100 randomly chosen abstracts which are covered by BRENDA but not by KID show a kinetic expression missing in the abstract as the main reason (61 times; see additional file 7). 35 times there is no abstract available for the given PubMed ID and in 4 cases the given expression was not contained in the dictionary of K_M_-expressions. Hence extending the algorithm to use whole articles instead of abstracts might improve its performance.

A minimum of useful information in terms of enzyme kinetics is available, if e.g. a kinetic expression and its numerical value can be linked to an enzyme (up to 133,774 times or 26% compared to the total number of database entries; see figure 4) or a ligand (up to 169,490 times or 33%). More information is attained by linking both categories together (up to 91,870 times or 18%).

### Calculation time

The developed algorithm allowed a very low calculation time compared to other text mining algorithms, with a per-abstract-calculation time of about 4 milliseconds per kilobyte text when acting on PubMed abstracts. Similar algorithms like e.g. BioRAT [19] and SUISEKI [20] require 3 to 5 seconds and 0.2 to 0.3 seconds per abstract, respectively, which would lead to a calculated processing time of approx. 600 to 1,000 days and 40 to 60 days, respectively for the amount of 16,953,021 abstracts. The velocity of the algorithm is based on the hash based structure of the dictionary used during identification, which in most cases ensures that each word needs to be treated once, except when the algorithm is forced to fall back when no kinetic flag is found at the last token (compare figure 1). Since the identification of numbers via regular expressions is only applied when a kinetic expression is detected, the increase of time of about one order of magnitude can be neglected when taking into account that this procedure is applied in approx. 3% of the sentences.

### Range and coverage of the extracted data

The classification of the K_M_, K_i _and K_d _by their numerical values give a clear and unbiased indication of the preferential range for each of these values, which in case of K_M _and K_d _fit into the expected range [28-30].

The classification of organisms contained within the results exhibits a clear majority for animals and bacteria, while plants are represented in smaller amounts. The spreading of the highest amount of single organisms over all categories indicates, that human and rat are the "hot spots" of scientific research.

## Conclusions

The short overall calculation time of the KID text mining algorithm and the resulting database prove evidence, that the presented algorithm can be a helpful tool for the annotation and collection of data for other databases like BRENDA.

"KID the KInetic Database" is a valuable help in the field of chemical and biological kinetics. The extent generated by a comprehensive text mining algorithm is comparable to that of databases with manually collected content and provides a reasonable quality marked by its precision and recall. Its major task is to accelerate the research by providing the scientist a large amount of data via its easy searchable web service, so that there is less need to consult written literature.

The approach described in here would be usable for the interpretation of whole publications, not just abstracts (with the exception of tables, which require a separate interpretation). Furthermore, information about enzymes is not restricted to their kinetic character and a further extension for categories to search is conceivable in order to attain even more data about an enzyme.

## Availability and requirements

The extracted information is available via the free web service named "KID the KInetic Database http://kid.tu-bs.de. The implementation of the algorithm is available on request from the authors.

## Competing interests

The authors declare that they have no competing interests.

## Authors' contributions

SH developed the text mining algorithm, with which the KID database has been created, and analysed and validated the data. BT generally consulted and helped to analyse and validate the data. DS consulted and supervised the whole work. All authors read and approved the final manuscript.

## Supplementary Material

Additional file 1 **Distribution of types of linkage**. In the file the amounts of results are subdivided into the category, the kinetic category and the type of linkage used. There are also tables included how many entries are found to the right and to the left of the kinetic value. Click here for file

Additional file 2 **Total amounts of results for single categories**. The file contains the total amounts of entries in the database subdivided into the categories and the available kinetic categories. Click here for file

Additional file 3 **Amounts of results found for combinations of categories**. This file comprises the amounts of results found for all possible combinations of categories subdivided into the kinetic categories. Click here for file

Additional file 4 **PubMed IDs taken for manual revision**. This file contains the PubMed IDs taken for the manual revision of the database and for calculating precision and recall. Click here for file

Additional file 5 **Precision in relation to linkage**. This file contains the classification of information contained in 1,002 entries from 510 randomly chosen abstracts in relation to the kind of linkage performed. Click here for file

Additional file 6 **Evaluation 100 randomized PubMedIDs covering KM in KID and not in BRENDA**. This file contains an evaluation of 100 randomized PubMedIDs covering K_M _which appear in KID but not in BRENDA. Click here for file

Additional file 7 **Evaluation 100 randomized PubMedIDs covering KM in BRENDA and not in KID**. This file contains an evaluation of 100 randomized PubMedIDs covering K_M _which appear in BRENDA but not in KID. Click here for file
